# Information Extraction Network Based on Multi-Granularity Attention and Multi-Scale Self-Learning

**DOI:** 10.3390/s23094250

**Published:** 2023-04-25

**Authors:** Weiwei Sun, Shengquan Liu, Yan Liu, Lingqi Kong, Zhaorui Jian

**Affiliations:** 1 College of Information Science and Engineering, Xinjiang University, Urumqi 830046, China; jakesun2020@stu.xju.edu.cn (W.S.);; 2 Xinjiang Multilingual Information Technology Laboratory, College of Information Science and Engineering, Xinjiang University, Urumqi 830046, China

**Keywords:** nested named entity identification, entity relationship extraction, machine reading comprehension, multi-grained attention mechanism, multi-scale self-learning mechanism

## Abstract

Transforming the task of information extraction into a machine reading comprehension (MRC) framework has shown promising results. The MRC model takes the context and query as the inputs to the encoder, and the decoder extracts one or more text spans as answers (entities and relationships) from the text. Existing approaches typically use multi-layer encoders, such as Transformers, to generate hidden features of the source sequence. However, increasing the number of encoder layers can lead to the granularity of the representation becoming coarser and the hidden features of different words becoming more similar, potentially leading to the model’s misjudgment. To address this issue, a new method called the multi-granularity attention multi-scale self-learning network (MAML-NET) is proposed, which enhances the model’s understanding ability by utilizing different granularity representations of the source sequence. Additionally, MAML-NET can independently learn task-related information from both global and local dimensions based on the learned multi-granularity features through the proposed multi-scale self-learning attention mechanism. The experimental results on two information extraction tasks, named entity recognition and entity relationship extraction, demonstrated that the method was superior to the method based on machine reading comprehension and achieved the best performance on the five benchmark tests.

## 1. Introduction

**Figure 1:**
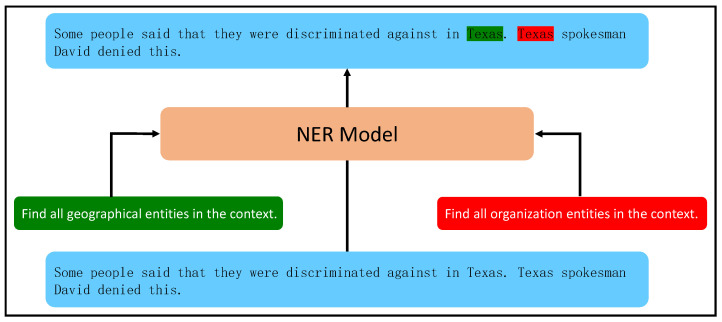
The process of recognizing entities by the named entity recognition model based on machine reading comprehension framework.

The information extraction task is a critical component of natural language processing, aimed at extracting structured knowledge from unstructured text [1,2,3,4,5]. Recently, pre-trained language models (PLMs) [6,7,8,9,10,11,12,13,14] have led to significant advancements in two classic information extraction tasks: named entity recognition (NER) and relation extraction (RE). In their work, Li et al. [15,16] described these tasks as requiring an understanding of context information and the ability to answer questions (queries). First, the corresponding queries are constructed based on entity or relationship types. Next, queries and context are connected and representations are learned using PLMs. Finally, the labels of each token are predicted, resulting in good performance on NER and RE tasks. Figure 1 illustrates the process of the model based on machine reading comprehension in identifying named entities.

**Figure 2:**
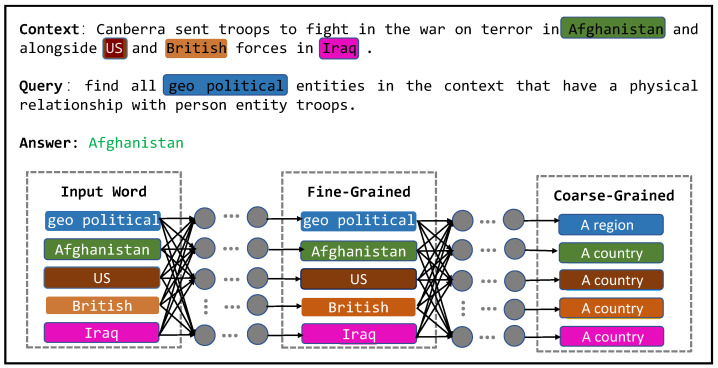
The information expressed by the hidden features of words has changed from fine-grained representation to coarse-grained representation.

Although joint encoding of query and context can capture inter-dependencies across the entire sequence, in a deep learning setting, the hidden features of words become increasingly similar with the increase of the encoder layers. This means that the hidden features no longer represent individual word information and become a fuzzy concept [17], as illustrated in Figure 2. Additionally, generating queries through crowdsourcing incurs significant human and time costs and has poor portability. Therefore, many researchers have transformed query generation into a template-filling problem. Examples of generating queries for different entity types are shown in Table 1, and examples of generating queries for different relationship types and entity types are shown in Table 2. When the query is long, connecting the query and context will cause the proportion of the context to relatively decrease. Therefore, as the query length increases, the information of individual words in the context will be diluted by the information of other words, resulting in the loss of some original information.

To address the issues discussed earlier, we propose a multi-granularity attention network. Our approach involves first independently encoding the query and context using dual encoders. Next, we employed a bidirectional attention network to capture word-level interactions between the query words and context words from two dimensions. We then used long-term-memory-gated attention to filter the original text information, preserving the source text representation of words relevant to the task. Even if the same phenomenon occurs due to the excessive number of encoding layers and the loss of original text information due to the query length, our model can still learn fine-grained features of individual words and retain the original text representation through long-term-memory-gated attention, thus enhancing its ability to read and understand text information. Finally, we combined the aforementioned components with the bidirectional attention vectors to construct a multi-granularity attention network. To the best of our knowledge, there is currently no work that uses dual encoders to solve the NER and RE tasks.

**Figure 3:**
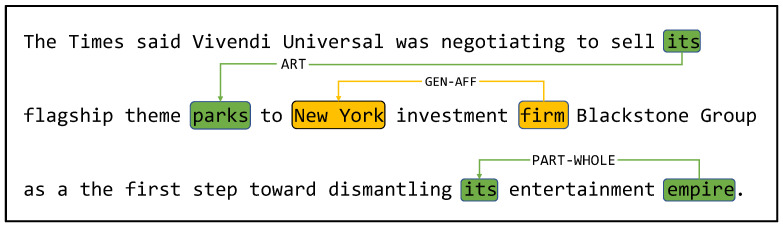
Generation of a candidate relationship triple. Under the action of the multi-scale self-learning mechanism, MAML-NET can extract not only the triples connected by yellow lines, but also the triples connected by two green lines. Without the effect of the multi-scale self-learning mechanism, only triples of yellow lines can be extracted.

In order to better help the model understand text and extract information for downstream tasks, a representation method that can describe and highlight the characteristics of a sequence can be relatively more effective. To this end, inspired by the human reader’s reasoning process of revisiting specific paragraphs or questions after single-round comprehension, we propose a self-learning mechanism that, with the guidance of the learning mechanism, focuses on different parts of the document each time from the perspectives of overall meaning and finer details and effectively collects evidence from the entire article to match the answer. As shown in Figure 3, MAML-NET can extract relationship triplets (its, ART, parks), (firm, GEN–AFF, New York), and (empire, PART–WHOLE, its) through the learning mechanism, by simulating the reasoning process of human readers. Specifically, we established a fusion module for interactive features and deep contextual semantic features, combining multi-head self-attention with depthwise separable convolution. At the same time, from both global and local perspectives, we used interactive information and deep contextual information to dynamically refine feature representations that match themselves. For global self-learning, we used self-attention to capture it; for local self-learning, we used deep separable multi-scale convolution, which is computed in parallel with self-attention. Overall, our proposed self-learning mechanism can effectively highlight key features in the text and help the model better understand and extract information for downstream tasks.

In summary, our contribution is as follows:

• A new information extraction method based on a reading comprehension model is proposed, which explores and utilizes the multi-granularity representation of the source sequence, encourages the model to fully utilize the interaction information and original information between the text and query, and enhances the expression ability of reading comprehension, thereby improving the information extraction capability.

• In order to dynamically collect evidence for candidate answers from multi-granularity text representations, a multi-scale self-learning network is proposed to refine semantic representations from global and local perspectives.

## 2. Related Work

### 2.1. Entity and Relation Extraction

Entity extraction, also known as named entity recognition, was initially approached using rule-based methods, which were expensive to build [18]. In recent years, deep learning has gained attention in various fields, and various neural network models have been proposed for this task. For example, Straková et al. [19] treated the nested NER as a sequence-to-sequence problem. Luan et al. [20] proposed a general information extraction method using dynamically constructed span graphs that share span representations. Li et al. [15] transformed the NER task into a machine reading comprehension task, where each entity type’s feature is a natural language query and entities are extracted by answering these queries (extracting two different categories of overlapping entities requires answering two separate questions).

Traditional entity relation extraction systems use a pipeline approach [21]. First, an entity extraction model identifies entities in the text. Then, a relation extraction model predicts the relations between the identified entities. Although this separate learning method makes processing the two sub-tasks more flexible, it ignores their inherent interaction and is severely affected by error propagation. To mitigate this problem, later work proposed joint learning methods. Early joint learning methods were based on crowdsourcing to build features, which was expensive. In subsequent research, several neural-network-based methods were proposed.

For example, Miwa et al. [22] proposed an end-to-end method that uses a tree-shaped LSTM model with parameter sharing to extract entities and relations. To better model the semantic relationship between entities, Katiyar et al. [23] improved upon this method using an attention mechanism to address the error propagation problem caused by the use of dependency trees. Zhang et al. [24] developed an end-to-end relation extraction model based on global optimization and used new LSTM features to better represent context. They also proposed a new method to integrate syntax information to facilitate global information learning. Sun et al. [25] proposed a graph convolutional network that operates on the entity relation bipartite graph. By introducing binary relation classification tasks, the bipartite graph structure of entity relations can be more interpretable and effectively utilized. At the same time, they pointed out that existing joint learning methods can predict entity spans correctly, but perform poorly when predicting entity types. Fu et al. [26] proposed a GraphRel end-to-end relation extraction model that uses graph convolutional networks (GCNs) to jointly learn named entities and entity relations. Zhang et al. [24] proposed a soft pruning model that can automatically learn how to select relevant substructures to focus on for relation extraction tasks. Shen et al. [27] proposed a triggered memory flow framework to enhance the interactive capabilities of entity recognition and relation extraction. Li et al. [16] viewed the relation extraction task as a multi-turn QA task. Zhao et al. [28] addressed the issue of a single query not being able to fully describe the meaning of entities and relations due to semantic diversity in multi-turn QA tasks. They designed a model that introduces diversity QA mechanisms and two answer selection strategies to integrate different answers.

### 2.2. NLP Tasks Based on the MRC Approach

Recently, there has been a trend of converting non-reading comprehension tasks in NLP into machine reading comprehension tasks. For example, Levy et al. [29] first converted the relation extraction task into the form of reading comprehension, which could adapt to zero-shot scenario problems. Mccann et al. [30] proposed a universal framework based on MRC, formalizing ten tasks such as question answering, sentiment analysis, and machine translation into question-answering tasks. Li et al. [15] proposed a unified MRC framework and applied it to the named entity recognition task. Yang et al. [31] proposed a label-aware enhanced language representation based on MRC and applied it to the named entity recognition task and time detection task. Du et al. [32] converted the event extraction task into a question task. Recent work has proposed a method of converting entity and relation extraction tasks into question-answering tasks. Although inspired by Li et al. [16], our work has the following characteristics: firstly, unlike the former using joint encoding to encode the query and context, MAML-NET independently encodes them through two pre-trained language model encoders. Secondly, MAML-NET uses bidirectional attention to establish the word-level interaction between the query and context and uses the original long-term memory gate attention to retain the important original information. Finally, through the original multi-scale self-learning network, it further understands deeper semantic information between the query and context. With the help of these features, MAML-NET’s performance was significantly improved.

## 3. Methodology

### 3.1. Overview

**Figure 4:**
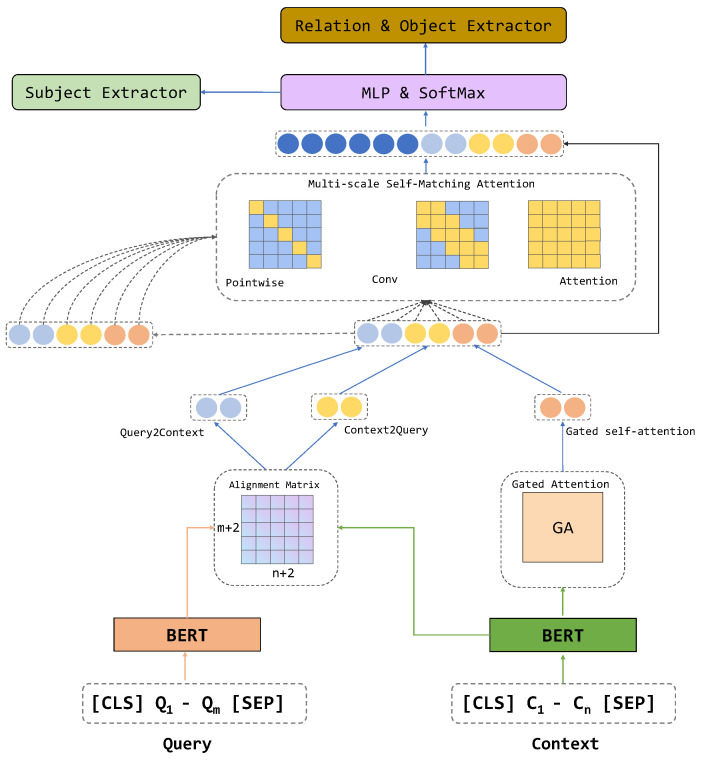
Overview of multi-granularity attention multi−scale self−learning network.

The multi-granularity attention and multi-scale self-learning network, as shown in Figure 4, consists of three main parts: (1) transforming the dataset of a specific task into a reading-comprehension-style dataset; (2) independently encoding the query and context using a Transformer-based encoder and establishing a multi-granularity representation of the source sequence; (3) passing the multi-granularity feature representation of the source sequence through the multi-scale self-learning network to further enhance the potential for reading comprehension at both the global and local levels.

### 3.2. Formalization of Tasks

Given a context sequence $x_{i}=\left(w_{1},w_{2},\ldots ,w_{n}\right)$ from the training set *D*, our task was to predict the relation between entities based on the information in each $x_{i}$, where each entity is assigned a label $y^{e}\in Y^{e}$ and each relation is assigned a label $y^{r}\in Y^{r}$. $Y^{e}$ is a set of entity types (e.g., ORG, GPE, etc.), and $Y^{r}$ is a set of relation types (e.g., ORG–AFF, PER–SOC, etc.). For the RE task, there may be multiple relations for a subject/object pair or the same entity may be shared between the subject and object, and we constructed a relation–object query for each subject.

#### 3.2.1. Named Entity Recognition

Given an input sequence $x_{i}$, identify all candidate spans $E_{i}=\left(w_{i},w_{j}\right)\in x_{i}$, and assign a label $y^{e}\in Y^{e}$ to each candidate span. Our goal was to maximize the likelihood of the training set *D*:

(1) $$\begin{array}{cc} \; & \prod_{i=1}^{\left|D\right|}\prod_{\left(w_{i},w_{j}\right)\in E_{i}}p\left(\left(w_{i},w_{j}\right)|x_{i},q\right)\\ = & \prod_{i=1}^{\left|D\right|}\left(\prod_{w_{i}\in E_{i}}p_{\mathrm{start}\;}\left(w_{i}|x_{i},q_{s}\right)\prod_{w_{j}\in E_{i}}p_{\mathrm{end}\;}\left(w_{j}|x_{i},q_{s}\right)\right) \end{array}$$

where $p_{start}\left(w_{i}|x_{i},q_{s}\right)$ and $p_{end}\left(w_{j}|x_{i},q_{s}\right)$ represent the probabilities of the start and end span positions for any $y^{e}\in Y^{e}$ category corresponding to $x_{i}$.

#### 3.2.2. Entity Relation Extraction

The goal was to maximize the data likelihood of the training set *D* for a given input sequence $x_{i}$ and a set of potential relationship triplets $T_{i}=\left(s,r,o\right)$:

(2) $$\begin{array}{cc} \; & \prod_{i=1}^{\left|D\right|}\prod_{\left(s,r,o\right)\in T_{i}}p\left(\left(s,r,o\right)|x_{i},q\right)\\ = & \prod_{i=1}^{\left|D\right|}\left(\prod_{s\in T_{i}}p_{s}\left(s|x_{i},q_{s}\right)\prod_{\left(r,o\right)\in T_{i}|s}p_{o}\left(\left(r,o\right)|s,x_{i},q_{o}\right)\right)\\ = & \prod_{i=1}^{\left|D\right|}\left(\prod_{s\in T_{i}}p_{s}\left(s|x_{i},q_{s}\right)\prod_{\left.o\in T_{i}\left|\right|s,r\right)}p_{o}\left(o|s,r,x_{i},q_{o}\right)\right) \end{array}$$

where *s*, *r*, *o*, $q_{s}$, and $q_{o}$, respectively, represent the subject, relation, object, and queries constructed based on the types of subjects and objects. $T_{i}|s$ denotes the set of relation triplets guided by subjects. $T_{i}|\left(s,r\right)$ denotes the object set guided by (subject,object) pairs. Firstly, a subject classifier $p_{s}\left(s|x_{i},q_{s}\right)$ is learned, which is used to identify all head entities in $x_{i}$. It is important to note that this head entity may not necessarily have a corresponding object and relation, i.e., the head entity may not be the subject. For each relation *r*, an object classifier $p_{o}\left(o|\left(s,r,x_{i},q_{o}\right)\right)$ is learned, which is used to identify the object corresponding to a specific relation for a given subject. Thus, the relation extraction task is formalized as a classification problem of identifying the subject and the object corresponding to a specific relation.

### 3.3. Input Layer

Given a query and context, we used $X^{q}\in R^{m}$ and $X^{c}\in R^{n}$ to represent the tokenized input of the query and context, respectively. They are independently mapped to a standard pre-trained bidirectional Transformer [33] for learning, resulting in the preliminary feature representations of the query and context: $O_{BERT}^{q}\in R^{(m+2)\times d}$ and $O_{BERT}^{c}\in R^{(n+2)\times d}$. The specific operations are shown in Formulas (3) and (4).

(3) $$O_{BERT}^{q}=BERT^{q}\left(X^{q}\right)$$

(4) $$O_{BERT}^{c}=BERT^{c}\left(X^{c}\right)$$

where *m* and *n* represent the lengths of the query and context sequences, respectively, and *d* represents the dimensionality of the BERT output.

### 3.4. Multi-Scale Attention

#### 3.4.1. Interactive Attention

To connect and integrate information from the context and query words, we employed a bidirectional attention flow network (BiDAF) [34]. This mechanism was designed to capture word-level interactions, where the interaction attention estimates the impact relationship between each query and context word.

The attention is calculated in two dimensions, from context to query and from query to context. Both attentions originate from the shared correlation matrix $U\in R^{\left(n+2)\times (m+2\right)}$, which is between the context $O_{BERT}^{c}$ and query $O_{BERT}^{q}$. $U_{ij}$ represents the correlation between the i-th context word and the j-th query word. The correlation matrix is calculated as follows:

(5) $$U_{ij}=W_{ij}\left[O_{BERT_{i}}^{c};O_{BERT_{j}}^{q};O_{BERT_{i}}^{c}\cdot O_{BERT_{j}}^{q}\right]$$

where $W_{ij}\in R^{3d}$ is a trainable weight matrix, · represents matrix multiplication, and ; represents matrix concatenation. We used *U* to compute attention vectors on both dimensions.

**Figure 5:**
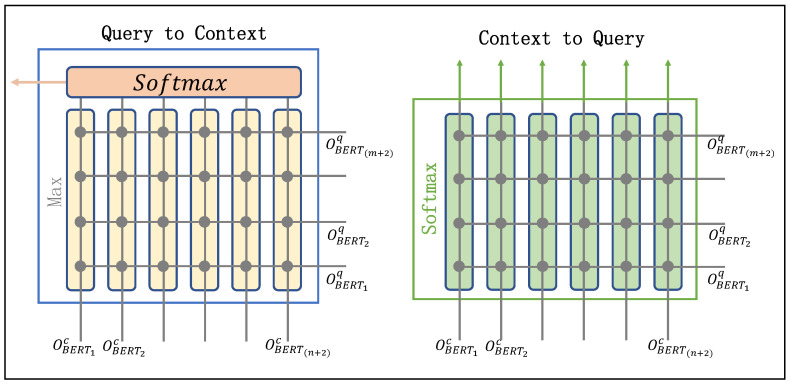
Diagram of interactive attention.

Query-to-context attention: The query-to-context (C2Q) attention mechanism measures the relevance between each context word and each query word, which is crucial for identifying entities. This attention mechanism simulates the process in which humans search for answers in text based on questions, enabling the model to selectively read the text based on the query. The specific structure is shown on the left side of Figure 5.

We calculated the attention score $s_{i}^{q_{c}}$ for each context word associated with the query. The specific operation is shown in Formula (6).

(6) $$s_{i}^{q_{c}}=\mathrm{softmax}\left(max\left(U_{i,:}\right)\right)$$

where $max(U_{i,:})$ represents the maximum value across the i-th row of the shared relevance matrix *U*. We then calculated the query aware context representing *r*. The specific operation is shown in Formula (7).

(7) $$O_{q2c}=\sum_{i=1}^{n+2}s_{i}^{q_{c}}\cdot O_{BERT}^{c}$$

Context-to-query attention The context-to-query (C2Q) attention represents the correlation between each query word and all words in the context. This attention mechanism aims to simulate the process of human beings reviewing questions after reading the text, thereby enabling the model to better understand the query: what the question is about. The specific structural system is shown on the right of Figure 5.

The attention score $s_{i}^{c_{q}}\in R^{m+2}$ represents the relevance of each context word to the query words, as shown in Formula (8).

(8) $$s_{i}^{c_{q}}=\mathrm{softmax}U_{i,:}$$

Then, we generated the context-aware query representation $O_{c2q}\in R^{(n+2)\times d}$, as shown in Formula (9).

(9) $$O_{c2q}=\sum_{i=1}^{m+2}s_{i}^{c_{q}}\cdot O_{BERT}^{q}$$

Finally, we concatenated the attention vectors from both dimensions and used this as the final interaction attention representation $O_{IA}\in R^{(n+2)\times d}$, as shown in Formula (10).

(10) $$O_{IA}=\left[O_{c2q};O_{q2c}\right]$$

#### 3.4.2. Long-Term-Memory-Gated Attention

**Figure 6:**
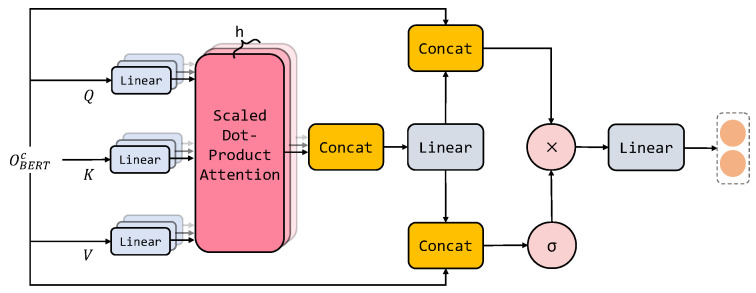
A description of long-term-memory-gated attention.

As deep neural networks are prone to homogeneity and the loss of the original information, we decided to incorporate the original information into the output results to reduce the information loss and achieve long-term memory. Therefore, we designed a long-term-memory-gated attention mechanism to allow the model to remember the original information of the context while controlling the flow of the context original information, capturing the source sequence features relevant to the task. The specific architecture is shown in Figure 6.

Long-term-memory-gated attention ($O_{LTMGA}\in R^{(n+2)\times d}$) is calculated as Formulas (11)–(15).

(11) $$O_{LTMGA}=O_{S}⊙O_{C}$$

(12) $$O_{C}=\sigma \left(W_{s}^{f}\left[O_{BERT}^{C};S_{MHSA}\right]\right)$$

(13) $$S_{MHSA}=\mathrm{softmax}\left(\frac{Q\cdot K^{T}}{\sqrt{d_{k}}}\right)\cdot V$$

(14) $$Q,K,V=f(O_{BERT}^{c})$$

(15) $$f\left(O_{BERT}^{c}\right)\left\{\begin{array}{c} Q=W_{q}\cdot O_{BERT}^{c}+b_{q}\\ K=W_{k}\cdot O_{BERT}^{c}+b_{k}\\ V=W_{v}\cdot O_{BERT}^{c}+b_{v} \end{array}\right.$$

where *Q*, *K*, and *V* are abstract matrices projected from the initial embedding matrix $O_{BERT}^{c}$ of the context. $s_{MHSA}$ is based on multiple scale-dot attention with 12 attention heads. $W_{s}^{f}\in R^{d\times 2d}$ represents trainable parameters. σ is an elementwise sigmoid function used to control the flow of information. ⊙ denotes elementwise multiplication.

Finally, the long-term-memory-gated attention flow $O_{LTGA}$ is aggregated with the interaction attention representation $O_{IA}$, and the result is normalized through layer normalization, resulting in the multi-granularity representation $O_{MGA}\in R^{(n+2)\times 3d}$. The process is shown in Formulas (16) and (17).

(16) $$O_{IA_{linear}}=Linear\left(O_{IA}\right)$$

(17) $$O_{MGA}=LN\left(O_{IA_{linear}}⊕O_{LTGA}\right)$$

where Linear denotes a linear transformation and ⊕ denotes vector addition. LN represents layer normalization.

### 3.5. Multi-Scale Self-Learning Layer

Although using the multi-granularity representation $O_{MGA}$ can enhance the representation of the source sequence by incorporating different levels of views, the enhanced source representation is still limited in understanding the information in the context, as a candidate answer typically contains information from multiple windows. In the constructed reading-comprehension-style relation extraction dataset, we enabled the model to learn from itself using the multi-granularity representation $O_{MGA}$, based on the multi-granularity information contained in $O_{MGA}$, to understand the overall idea of the context and important details related to the task. To achieve this, we designed two strategies to enable the model to self-learn at different scales.

#### 3.5.1. Global-Information-Gated Attention

Although query-to-context can allow the model to selectively read text with a question in mind and context-to-query can allow the model to read text more broadly and deepen its understanding of the query, this is clearly not enough because just reading selectively and broadly will make it difficult to understand the true meaning of the text. Therefore, we hope to use this strategy to let the model review the entire text again and clarify the main idea of the text. For a given $O_{MGA}$, it is first mapped to three representations, and then, the final representation $G_{att}\in R^{(n+2)\times d}$ is obtained. The specific operation is shown in Formulas (18) and (19).

(18) $$LMGS\left(O_{GMA}\right)=\mathrm{softmax}\left(\frac{Q\cdot K^{T}}{\sqrt{d_{k}}}\right)\cdot V_{1}$$

(19) $$V_{1}=W_{v}V_{1}$$

where $Q,K,V_{1}=Linear_{1}\left(O_{GMA}\right)$, $Linear_{2}\left(O_{GMA}\right)$, $Linear_{3}\left(O_{GMA}\right)$, and $W_{V}$ is the projection parameter.

#### 3.5.2. Local-Features-Focused Attention

In order to simulate human reading habits, after full-text scanning, question-based reading, and overall in-depth reading, we hope that the model can learn the key information related to the query in the text through its own understanding, rather than just relying on the query. To achieve this, we used convolutional operations to capture more refined local features, so that the model can grasp the details related to the task. We chose a deep convolution with pointwise projection and context transformation characteristics for the convolutional operations. We chose one variant of deep convolution, dynamic convolution, for the operation. Each convolutional submodule in the dynamic convolution contains convolutional kernels of different sizes, and it is called multi-scale dynamic convolution because of the different scales of the convolutional kernels. The convolutional kernel size *k* is calculated as shown in Formulas (20) and (21).

(20) $$Conv_{k}\left(O_{GMA}\right)=W_{out}MSConv_{k}\left(V_{2}\right)$$

(21) $$V_{2}=W_{v}O_{GMA}$$

where $W_{V}$ and $W_{out}$ are learnable hyperparameters and $W_{V}$ is a pointwise projecting transformation matrix. The projection operation performed on the input of multi-scale dynamic convolution $V_{2}$ is the same as $V_{1}$, and MSConv represents the multi-scale dynamic convolution. The local self-learning sequence (LS) in the output of $O_{GMA}$ through multi-scale dynamic convolution is $LS\in R^{(n+2)\times d}$. The specific operation is shown in Formulas (22) and (23).

(22) $$LS_{i,c}=MSConv_{k}\left(O_{GMA}\right)$$

(23) $$MSConv_{k}\left(O_{GMA}\right)=\sum_{j=1}^{k}\left(\mathrm{softmax}\left(\sum_{c=1}^{d}W_{j,c}^{Q}O_{GMA_{i,c}}\right)\cdot O_{GMA_{i+j-\left\lceil \frac{k+2}{2}\right\rceil ,c}}\right)$$

where *d* is the hidden layer size.

Shared projection: To represent the context sequence in the same hidden space, we shared the projections $V_{1}=W_{V}V_{1}$ and $V_{2}=W_{V}O_{GMA}$. By sharing the projections, the global and local self-attention representations are mapped to the same hidden space. If two different projection matrices $W_{1}$ and $W_{2}$ are used, it is referred to as independent projection, i.e., $V_{1}=W_{V_{1}}V_{1}$.

Dynamically selected convolutional kernels: In order to dynamically and autonomously select the weights of the convolutional kernels of different scales, a gating mechanism was used. The specific operation is shown in Formula (24).

(24) $$\mathrm{Conv}\left(O_{GMA}\right)=\sum_{i=1}^{n}\frac{\exp\left(a_{i}\right)}{\sum_{j=1}^{n}\exp\left(a_{j}\right)}{\mathrm{Conv}}_{k_{i}}\left(O_{GMA}\right)$$

Then, we fused the global and local self-learned features to obtain the final label prediction vector $O_{T}\in R^{(n+2)\times d}$, which was achieved through the following operation.

After the fusion of the global self-learning feature and the local self-learning representation, the final label prediction vector $O_{T}\in R^{(n+2)\times d}$ is obtained. The specific operation is shown in Formula (25):

(25) $$O_{T}=LS⊕GS$$

where ⊕ represents matrix addition operation.

### 3.6. Decoding Layer

In the relation extraction task, we formalized the task as a query-based labeling problem. The softmax layer takes the label prediction vector $O_{T}$ through a multilayer perceptron and calculates the normalized probabilities of the BIOES entity labels, thus transforming the binary task of predicting the start and end indices into a five-classification task.

During training, we jointly trained the two stages of the objective function: the multiple answer task for subject extraction and the single-answer task for joint relation and object extraction. The two tasks are cascaded using a parameter-sharing strategy. The objective function is calculated as shown in Formulas (26)–(28).

(26) $$L_{re}=\lambda L_{subject}+\left(1-\lambda \right)L_{\left(relation,object\right)}$$

(27) $$L_{subject}=-\sum_{s\in T_{i}}\log p_{s}\left(s|x_{i},q_{s}\right)$$

(28) $$L_{relation,object}=-\sum_{o\in T_{i}|\left(s,r\right)}\log p_{o}\left(o|s,r,x_{i},q_{o}\right)$$

where ∈[0,1] is a hyperparameter that controls the weighting between the two objective functions. The experimental results showed that an α of 0.25 achieved a good balance and led to a good performance.

In the nested named entity recognition task, we used two n-class classifiers to predict the start and end indices. The objective function was calculated as shown in Formulas (29)–(32).

(29) $$L_{ner}=L_{start}+L_{end}+\alpha L_{match}$$

(30) $$L_{match}=-\sum_{w_{i},w_{j}\in E_{i}}P_{match}\left(p_{start},P_{end}\right)$$

(31) $$L_{start}=-\sum_{w_{i}\in E_{i}}p_{start}\left(w_{i}|x_{i},q_{s}\right)$$

(32) $$L_{end}=-\sum_{w_{j}\in E_{i}}p_{end}\left(w_{j}|x_{i},q_{s}\right)$$

where $L_{match}$, $L_{start}$, and $L_{end}$ represent the losses for start–end matching, start prediction, and end prediction, respectively. α∈[0,1].

## 4. Experimental Section

### 4.1. Datasets

For the NER task, we conducted experiments on the ACE2004, ACE2005, and GENIA datasets. To ensure fairness, we followed the same data splitting as Yang et al. [35] and Chen et al. [36] for the ACE2004 and ACE2005 datasets, and followed Katiyar et al. [37] for the the GENIA dataset, which split the training, development, and test sets into a ratio of 8.1:0.9:1.0.

For the entity relation extraction task, we conducted experiments on the ACE2004 and ACE2005 relation datasets, which were collected from various domains such as news agencies and online forums by the Linguistic Data Consortium. To ensure fairness, we followed the same data splitting as Luan et al. [20], who split the ACE2005 dataset into training, development, and test sets and performed 5-fold cross-validation on the ACE2004 dataset.

### 4.2. Experimental Setups

We developed MAML-NET using Python and PyTorch. Two independent BERT-base-uncased [6] were used to encode the query and context. During training, AdamW [38] was used as the optimizer, with a learning rate of $2\times 10^{-5}$, 20 epochs, and a batch size of 30. To regularize the model, different dropout rates were applied to different layers of MAML-NET, with the best setting ranging from 0.2 to 0.4. In addition, MAML-NET uses an early stopping strategy based on the validation loss. If the validation loss does not improve over the last 5 epochs, training will be terminated early. It should be noted that, on the GENIA dataset, biobert-base-cased-v1.2 [39] was used for encoding.

### 4.3. Named Entity Recognition

#### 4.3.1. Baseline Models for Named Entity Recognition

(1) Straková et al. [23] viewed nested NER as a sequence-to-sequence problem, where each token in the input sequence is assigned to its corresponding entity and a recursive neural network is used to capture the nested entity structure. (2) Luan et al. [20] proposed a general method for information extraction using dynamically constructed span graphs to share span representations. By representing all possible entities as a dynamically constructed graph, entity representations can be shared and relationships between entities can be captured. (3) Li et al. [15] transformed the NER task into a QA task by treating named entities as questions and using the context as answers. By training the model with QA data during training, NER can be performed without labeled entities. (4) Yu et al. [40] used the idea of dependency tree parsing of graphs to score the beginning and ending tokens of entities in a sentence and used these scores to determine the boundaries of entities. (5) Hou et al. [41] injected semantic type word embeddings into entity embeddings to reduce differences in contextuality. This can help the model better distinguish entities and improve the performance of NER.

#### 4.3.2. Experimental Results of Named Entity Recognition

The Table 3 shows the performance of MAML-NET on the ACE04, ACE05, and GENIA datasets relative to the previous state-of-the-art methods. Compared with the baseline model, MAML-NET improved the F1-score by 0.12%, 0.18%, and 0.32%, respectively. We speculate that this is because MAML-NET fully utilizes context and query information, not only to learn prior knowledge of named entities through the query, but also to exploit the interaction between the query and context, i.e., selectively reading the context based on the query and deepening the understanding of the query through reading the text. In addition, the model can alleviate the problem of information loss in the original sequence through the long-term-memory-gated attention mechanism. Finally, by repeatedly comprehensively grasping and studying the details of the text, the model achieved excellent results. However, Li et al. [15] only transformed the NER task into an MRC task and then processed the problem through a general deep neural network, which can only obtain prior knowledge of entities, and it is difficult to analyze the details and interaction between the text and questions. Yu et al. [40] and Straková et al. [23] did not use the query to transform the NER task into the MRC task, but learned the text information through RNNs, and then, the decoder recognized the entities based on this deep text information. We believe that, although their methods can avoid the problem of losing the original text information due to long queries, they also cut off the possibility of the model learning prior knowledge of entities in the query. However, MAML-NET can not only learn the deep text information, but also effectively alleviate the problem of similar word hidden features through the long-term-memory-gated network. Although Yu et al. [40] fused fine-grained semantic information into entity embedding to reduce uniqueness, we believe that this fusion strategy is not necessarily better than the text–query interaction strategy adopted in this paper. The results showed that our multi-scale attention mechanism and multi-scale self-learning mechanism were effective for named entity recognition tasks.

### 4.4. Baseline Models for Relation Extraction

We compared our approach with the following baselines: (1) Zhang et al. [24] transformed the relation extraction task into a table-filling problem. (2) Miwa et al. [22] proposed the first joint entity relation extraction method and used tree-based LSTMs to capture dependency information. (3) Straková et al. [23] replaced the tree-based structure with attentional LSTMs. (4) Sun et al. [25] used graph convolutional networks to address the problem of exploring entity relations. (5) Li et al. [16] proposed an MRC-based method. (6) Zhao et al. [28] extended Li et al.’s [16] method by constructing multiple queries for each context. (7) Shen et al. [27] memorized the entity and relationship category information learned in the task through the constructed memory module.

#### Experimental Results of Relation Extraction

Table 4 shows the performance of MAML-NET compared to state-of-the-art methods on the ACE2004 and ACE2005 datasets. It can be observed that MAML-NET outperformed all baseline models in terms of entity and relation extraction performance on both datasets. Specifically, MAML-NET improved the F1-scores of entity and relation extraction by 4.3% and 9.8%, respectively, on the ACE2004 dataset and by 2.0% and 0.9%, respectively, on the ACE2005 dataset. This indicated that MAML-NET performed better on the RE task compared to the NER task. We speculated that this is because MAML-NET can learn both the prior knowledge of named entities and entity relations in RE task. Furthermore, due to the presence of error propagation, the advantage obtained in the entity recognition stage was not simply transmitted to the relation extraction task, but continued to expand. Additionally, the MRC framework with multiple queries performed better than the single-query framework. We believe that this was because the multi-query framework allowed the model to more finely divide the RE task, rather than simply being a vague task such as “Find all relation”. However, neither the single-query framework nor the multi-query framework for RE models achieved the same level of performance as MAML-NET. We speculated that, on the one hand, although MAML-NET is based on a single-query framework; due to the different query design strategies, the performance of MAML-NET’s single-query framework was not inferior to the multi-query framework. On the other hand, unlike other models that only use BERT to learn the interaction information between query and context, MAML-NET has a dedicated interaction module, which can better utilize the implicit association information in the query and context.

### 4.5. Ablation Study

In order to investigate which aspects improved the performance of our model, we conducted an ablation study on the ACE2005 relation extraction dataset to understand the impact of each module on the model. We can observe the following results from Table 5.

Multi-scale attention (MSM): When the multi-scale self-learning network (MSM) is removed, compared to MAML-NET, the F1-score for entity and relation extraction decreased by 1.1% and 2%, respectively. We believe this is because the multi-scale self-learning layer can not only help the model understand the text as a whole, but also enable the model to discover important details relevant to the task, ultimately allowing the model to dynamically collect evidence from the entire paragraph to answer the question, resulting in improved performance in entity and relation extraction.

Long-term-memory-gated attention (LTMGA): When the long-term-memory-gated attention flow (LTMGA) was removed, compared to MAML-NET, the F1-score for entity and relation extraction decreased by 0.6% and 1.7%, respectively. This is reasonable because, although the interaction between the query and context can provide complementary information to each other, it also causes some loss of the original context information. The introduction of our long-term-memory-gated attention flow can alleviate this phenomenon, resulting in improved performance in both entity and relation extraction.

Interactive attention (IA): When using interactive attention, compared to MAML-NET, the F1-score for entity and relation extraction decreased by 1.6% and 2.9%, respectively. Compared to the joint encoding method (-IA-MSM-LTMGA), the F1-score for entity and relation extraction decreased by 4.8% and 3.5%, respectively. We believe this is because interactive attention can selectively collect relevant representations of other words in the source sequence during the word-encoding process based on the similarity between words in the text and the question. This not only alleviates the problem of encoding information similarity between different words, but also increases the distinguishability of encoding information between dissimilar words.

### 4.6. Case Study

To demonstrate the effectiveness of the MAML-NET model in improving entity and relation extraction by enhancing the reading comprehension ability, we present the examples shown in Table 6.

Table 6 compares the output of our MAML-NET with the previous state-of-the-art reading-comprehension-based relation extraction model (Multi-QA). In the first example, Multi-QA was unable to determine the triplets (units, ORG–AFF, republican guard) and (units, PHYS, capital). In the second example, Multi-QA was unable to determine the triplets (outlet, GEN–AFF, Milton Keynes) and (outlet, PART–WHOLE, easy). In the third example, Multi-QA was unable to determine the triplets (forces, PHYS, home) and (microbiologist, ART, home). From these examples, we observed that MAML-NET performed much better than Multi-QA when the subject or object participates in multiple relationship triplets at the same time. We speculated that, on the one hand, in NER tasks, the existence of nested named entities may cause some named entities to be unidentifiable if the model cannot understand the text from both the global and local perspectives. On the other hand, for RE tasks, if the model ignores certain key details, some relationships between entities will not be identified. This also demonstrates that MAML-NET has a good ability to process global information and local features.

## 5. Conclusions

This article proposed a multi-scale self-learning network based on multi-granularity attention to address the issue of homogeneity that arises with the increasing number of network layers in RE tasks, as well as the problem of the loss of the text’s original information in MRC tasks. Firstly, to effectively utilize the unique query information in MRC tasks, we proposed a multi-granularity attention mechanism, which allowed the model to learn the interaction information between the context and query in the source sequence through bidirectional attention and retained the task-related original context information through long-term-memory-gated attention flow. Then, to enable the model to consider both the global information and local features of the text, we proposed a self-learning mechanism to further improve the model’s understanding of the context. Finally, we optimized the model through joint learning. The experiment showed that our model was effective in entity relation extraction tasks.

Future research can be conducted in the following directions. First, although the introduced query can provide prior knowledge helpful for the task, the knowledge provided was still limited. Therefore, knowledge graphs or other knowledge can be used as prior knowledge for data augmentation. Second, the model still lacks entity recognition and relation extraction in long sentences. Therefore, multi-hop reading comprehension methods can be used to address entity recognition and relation extraction tasks in long sentences.

## Figures and Tables

**Table 1:** Examples of generating queries based on program templates for different entity types. The blue font is the entity type to be filled.

Entity Type	Query
Facility (FAC)	find all facility entities in the context.
Geopolitical (GPE)	find all geopolitical entities in the context.
Location (LOC)	find all location entities in the context.
Organization (ORG)	find all organization entities in the context.
Person (PER)	find all person entities in the context.
Vehicle (VEH)	find all vehicle entities in the context.
Weapon (WEA)	find all weapon entities in the context.

**Table 2:** Examples of generating question queries based on program templates for different relationship types, where $e_{head}$ represents the header entity obtained through the entity extraction phase. The header entity is not necessarily a real subject.

Relation Type	Head Type	Tail Type	Query
ART	GPE	FAC	find all facility entities in the context that have an artifact relationship with geopolitical entity $e_{head}$.
ART	GPE	WEH	find all vehicle entities in the context that have an artifact relationship with geopolitical entity $e_{head}$.
PHYS	FAC	FAC	find all facility entities in the context that have a physical relationship with facility entity $e_{head}$.
PART–WHOLE	GPE	GPE	find all geopolitical entities in the context that have a part–whole relationship with geopolitical entity $e_{head}$.
PART–WHOLE	GPE	LOC	find all location entities in the context that have a part–whole relationship with geopolitical entity $e_{head}$.

**Table 3:** Comparison of the performance of ACE2004, ACE2005, and GENIA. † indicates the results we achieved through their code. Bold numbers indicate the best results.

ACE2004			
Model	P	R	F1
Straková et al. [19]	-	-	84.40
Luan et al. [20]	-	-	84.70
Yu et al. [40] †	85.42	**85.92**	85.67
Li et al. [15] †	86.38	85.07	85.72
MAML-NET	**86.82**	84.88	**85.84**
ACE2005			
Model	P	R	F1
Straková et al. [19]	-	-	84.33
Li et al. [15] †	85.48	84.36	84.92
Yu et al. [40] †	**84.50**	84.72	84.61
Hou et al. [41]	83.95	**85.39**	84.66
MAML-NET	85.26	84.95	**85.10**
GENIA			
Model	P	R	F1
Straková et al. [19]	-	-	76.44
Li et al. [15] †	79.62	76.8	78.19
Yu et al. [40] †	79.43	78.32	78.87
Hou et al. [41]	79.45	78.94	79.19
MAML-NET (ours)	**79.65**	**79.37**	**79.51**

**Table 4:** Performance comparison of ACE2004 and ACE2005. † and †† represent baselines based on the MRC framework, and †† represent baselines with multiple queries for the context. Bold numbers indicate the best results.

Dataset	Model	Entity			Relation		
		P	R	F	P	R	F
ACE2004	Miwa et al. [22]	80.8	82.9	81.8	48.7	48.1	48.4
	Straková et al. [23]	81.2	78.1	79.6	46.4	45.3	45.7
	Li et al. [16] †	84.4	82.9	83.6	50.1	48.7	49.4
	MAML-NET	**87.9**	**88.8**	**87.9**	**57.9**	**60.2**	**59.2**
ACE2005	Miwa et al. [22]	82.9	83.9	83.4	57.2	54.0	55.6
	Straková et al. [23]	84.0	81.3	82.6	55.5	51.8	53.6
	Zhang et al. [24]	-	-	83.5	-	-	57.5
	Sun et al. [25]	83.9	83.2	83.6	64.9	55.1	59.6
	Li et al. [16] †	84.7	84.9	84.8	64.8	56.2	60.2
	Zhao et al. [28] †	85.1	84.2	84.6	57.8	61.9	59.8
	Zhao et al. [28] ††	85.9	85.2	85.5	62.0	62.2	62.1
	Shen et al. [27]	86.7	87.5	87.6	62.2	**63.4**	62.8
	MAML-NET	**89.5**	**88.9**	**89.6**	**69.4**	58.8	**63.7**

**Table 5:** Ablation trials on ACE2005. Bold numbers indicate the best results.

Dataset	Model	Entity			Relation		
		P	R	F	P	R	F
ACE2005	MAML-NET	**89.5**	**89.8**	**89.6**	**69.4**	**58.8**	**63.7**
	-MSL	89.0	88.1	88.5	66.2	57.7	61.7
	-LTMGA	88.9	89.1	89.0	66.0	58.6	62.0
	-MSM&LTMGA	87.4	88.6	88.0	64.9	57.2	60.8
	-IA&MSM&LTMGA	84.7	84.9	84.8	64.8	56.2	60.2

**Table 6:** Comparing the MAML-NET model with multi-QA [16]. The triples in red represent the results extracted by MAML-NET.

Sentence 1	… US officials say some intelligence indicates a red line may have been drawn around the capital with republican guard units ordered to use chemical weapons once US and allied troops cross it. …
Multi-QA	((PER, units), ART, (WEA, weapons)); ((PER, troops), ORG–AFF, (GPE, US)).
MAML-NET	((PER, units), ART, (WEA, weapons)); ((PER, troops), ORG–AFF, (GPE, US)); ((PER, units), ORG–AFF, (ORG, republican guard)); ((PER, units), PHYS, (GPE, capital)).
Sentence 2	… The deadlock, and subsequent lack of any films, has been threatening to de-rail the debut of easy Cinema s first outlet in Milton Keynes, just north of London, which is due to open its doors on May 23. …
Multi-QA	((GPE, Milton Keynes), PHYS, (GPE, London)).
MAML-NET	((GPE, Milton Keynes), PHYS, (GPE, London)); ((ORG, outlet), GEN–AFF, (GPE, Milton Keynes); ((ORG, outlet), PART–WHOLE, (ORG, easy)).
Sentence 3	…And as part of that effort, US special forces today raided the home of the Iraqi microbiologist known as Dr. Germ. …
Multi-QA	((PER, forces), ORG–AFF, (GPE, US)); ((PER, microbiologist), GEN–AFF, (GPE, Iraqi)).
MAML-NET	((PER, forces), ORG–AFF, (GPE, US)); ((PER, microbiologist), GEN–AFF, (GPE, Iraqi)); ((PER, forces), PHYS, (FAC, home)); ((PER, microbiologist), ART, (FAC, home)).

## Data Availability

Not applicable.
